# A Hitchhiker's guide to humanized mice: new pathways to studying viral infections

**DOI:** 10.1111/imm.12906

**Published:** 2018-03-09

**Authors:** Jessica Katy Skelton, Ana Maria Ortega‐Prieto, Marcus Dorner

**Affiliations:** ^1^ Section of Virology Department of Medicine Imperial College London London UK

**Keywords:** Haematopoiesis, humanized mice, immune system, stem cell, viral

## Abstract

Humanized mice are increasingly appreciated as an incredibly powerful platform for infectious disease research. The often very narrow species tropism of many viral infections, coupled with the sometimes misleading results from preclinical studies in animal models further emphasize the need for more predictive model systems based on human cells rather than surrogates. Humanized mice represent such a model and have been greatly enhanced with regards to their immune system reconstitution as well as immune functionality in the past years, resulting in their recommendation as a preclinical model by the US Food and Drug Administration. This review aims to give a detailed summary of the generation of human peripheral blood lymphocyte‐, CD34^+^ haematopoietic stem cell‐ and bone marrow/liver/thymus‐reconstituted mice and available improved models (e.g. myeloid‐ or T‐cell‐only mice, MISTRG, NSG‐SGM3). Additionally, we summarize human‐tropic viral infections, for which humanized mice offer a novel approach for the study of disease pathogenesis as well as future perspectives for their use in biomedical, drug and vaccine research.

**Abbreviations**
AAV: adeno‐associated virus
AdV: adenovirus
BLT: bone marrow/liver/thymus
CMV: cytomegalovirus
DENV: dengue virus
EBV: Epstein–Barr virus
HIS: human immune system
HIV: human immunodeficiency virus
HLA: human leucocyte antigen
HSC: haematopoietic stem cell
HTLV: human T‐lymphotrophic virus
huPBL: human peripheral blood leucocyte
KSHV: Kaposi's sarcoma‐associated herpesvirus
MHC: major histocompatibility complex
SIRP*α*: signal regulatory protein *α*
ZIKV: Zika virus

## Humanized mouse models

Humanized mice have become an essential tool in validating infectious disease research in recent years. As affordable small animal models for studying basic research and translational medicine, the field is rapidly expanding and is accompanied by the demand for improved models with increased humanization and efficacy.

The study of many viruses *in vivo* requires the use of surrogate models (e.g. simian immunodeficiency virus and non‐human primates) or pathogen adaptation to non‐human systems (e.g. Ebola virus in mice^1^). Yet, the variation in viral species and host requirements makes these alternative models less suitable for studying virus–host interactions. The development of humanized mice allows the study of pathogens within their natural host cells, offering the affordability, accessibility and flexibility that other models cannot, making them a powerful tool for cutting‐edge biomedical and preclinical research.

However, despite the promising improvements observed in recent years, numerous aspects of immune system development within these models are still under‐represented or underdeveloped and the goal remains to create a completely physiological human immune response comprising all haematopoietic lineages, encompassing the functionality and correct proportions observed in a human. Hence, there is still much need for the advancement and development of current and novel humanized mouse models.

## HuPBL mice

**Figure 1:**
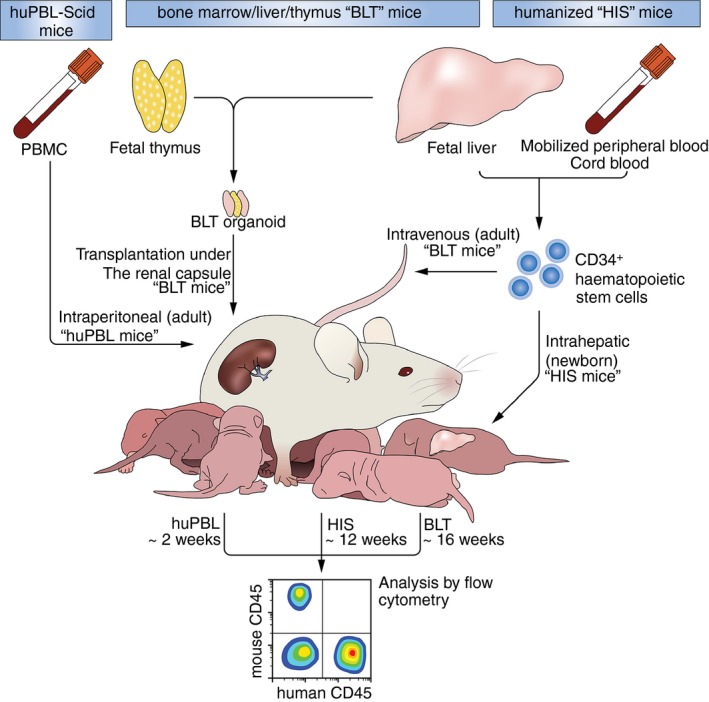
Generation of humanized mouse models, cellular origin, delivery routes and reconstitution times of huPBL, HIS and BLT mice.

The first humanized mouse developed in 1983 was the human peripheral blood lymphocyte (huPBL) mouse model, created via intraperitoneal injection of human peripheral blood lymphocytes into an immunodeficient mouse that may be exposed to a sub‐lethal dose of irradiation^2^ (Fig. 1). The lack of a fully functioning murine immune system facilitates the temporary circulation of human cells, particularly high levels of the completely functional, educated T‐cell populations in all major organs.

The huPBL model also facilitates engraftment of low levels of B cells and some antibody production can be observed, with immune memory maintained from the donor. Engraftment of other important haematopoietic lineages (e.g. myeloid derived cells) is not supported in this model because of the rapid expansion of T cells. Furthermore, the injected T cells maintain their education from the donor, resulting in their rapid activation as they recognize the murine cells, ultimately leading to the development of graft‐versus‐host disease making this model only suitable for short‐term studies.^3^

## HIS mice

The human immune system (HIS) mice use immunodeficient mouse strains injected with human CD34^+^ haematopoietic stem cells (HSC).^4^ most commonly derived from cord blood,^5^ fetal livers or granulocyte–macrophage colony‐stimulating factor (GM‐CSF) ‐mobilized peripheral blood (Fig. 1).^6^ Engraftment depends heavily on stem cell origin, injection route, HSC donor, background murine strain, irradiation status and engraftment age. Most haematopoietic lineages are engrafted, including several myeloid subsets. However, the major functionality of the T‐cell population is limited by the absence of human primary lymphoid organs, hence T cells are educated via murine major histocompatibility complex (MHC) class I and II molecules, stunting T‐cell development.

## *B*one marrow, *L*iver and *T*hymus (BLT) mice

Co‐xenotransplantation of autologous human fetal liver and thymic tissue under the murine renal capsule, alongside an intravenous CD34^+^ HSC injection, subverts many issues seen in HIS mice (Fig. 1). Primarily the human fetal thymic tissue allows the formation of a ‘BLT organoid’, which supports functional and educated T‐cell populations and boasts superior engraftment of all other major haematopoietic lineages.^7^ The engraftment of the HSC and the thymic transplant is aided by the addition of the fetal liver tissue. However, this liver tissue is not maintained in the mature organoid. Unlike previous models, which demonstrate a lack of human immune cell engraftment at mucosal surfaces, the BLT mouse model improves not only the cellular localization, dissemination and lymphoid nodule development accompanied by an increased colonization of lymphoid organs but also enhances reconstitution of the gastrointestinal and mucosal tracts.^7^ In addition to this, gut‐associated lymphoid tissues contain aggregates of human cells, functional IgA‐producing plasma cells and detectable levels of human IgG and IgM.^7^, ^8^ The BLT mouse model is a widely used tool for dissecting pathogen transmission, dissemination and pathogenesis, alongside analysis of vaccine candidates and microbicides.^9^, ^10^, ^11^

## Background strains

Small animal models are an affordable tool for answering a variety of biological questions regarding infectious diseases, genetic conditions, therapeutic testing and vaccine candidates. Humanized mouse models can be developed by using a highly immunodeficient background strain e.g. severe combined immunodeficiency (*scid*)^−/−^, *NOD.Cg‐scid* ^−/−^ (*NS*), *NOD.Cg‐scid* ^−/−^ *IL2Rγ* ^−/−^ (NSG), *NOD.CgRag1* ^−/−^ *IL2Rγ* ^−/−^ (NRG) and *NOD/Shi‐scid IL2rg* ^*−/−*^ (NOG) (Table 1). The use of protein kinase, DNA‐activated, catalytic polypeptide (*Prkdc*) knockout leads to defective adaptive immune cell development, yielding the *scid* phenotype.^2^ Similarly, the use of recombination activating gene (*Rag1/2*) knockouts severely impairs B‐cell and T‐cell receptor recombination and natural killer (NK) ‐cell development^12^ (Table 1). However, complications still arise with the residual murine myeloid cell compartment, which can still phagocytose non‐self human cells. To overcome this, many models include the NOD background, whose human signal regulatory protein‐*α* (SIRP*α*) allele has been shown to restrict murine macrophages from phagocytosing human cells through SIRP*α*/CD47 interaction.^13^ Alternatively, human SIRP*α* can also be expressed transgenically^14^, ^15^ (Table 2).

**Table 1:** Basic immunodeficient murine background strains for xenotransplantation

Name abbreviation	Strain name	Details	Reference
scid	*Prkdc* ^*scid−/−*^	Lacks B and T cells due to Prkdc deficiency	16
NOD.scid	*NOD.CB17‐Prkdc* ^*scid*^	Lacks B and T cells due to Prkdc deficiency	4, 17, 18
		NOD SIRPa gene aids in preventing murine phagocytosis of donor HSC	
NSG	*NOD.Cg‐*Prkdc^scid^ Il2rg^tm1WjI^	NOD SIRPa gene aids in preventing murine phagocytosis of donor HSC	19, 22
		Lacks B and T cells due to Prkdc deficiency	
		IL2rg deficiency prevents NK cell development	
NRG	*NOD.Cg‐Rag1* ^*tm1Mom*^ *Il2rg* ^*tm1Wjl*^	NOD SIRPa gene aids in preventing murine phagocytosis of donor HSC	23
		Rag1 deficiency prevents recombination and thus maturation of B and T cells	
		IL2rg^−/−^deficiency prevents NK cell development	
NOG	*NOD/Shi‐scid IL2rg* ^*−/−*^	NOD SIRPa gene aids in preventing murine phagocytosis of donor HSC	8, 24
		Contains non‐functional truncated IL2rg, resulting in stunted maturation of B and T cells	

HSC, haematopoietic stem cell; IL‐2, interleukin‐2; SIRPa, signal regulatory protein *α* gene.

**Table 2:** Cell subsets availability and functionality in humanized mice

Cell subset	Model	Present/absent	Reported improvements to engraftment and subset development	Advantages	Disadvantages	Reference
B cells	huPBL	+	–	Donor humoral repertoire transferred	Low levels of human B cells	19
					No *de novo* immune responses	
					Rapid development of GvHD	
	HIS	+	–	–	Low serum human immunoglobulin	25, 26
					Limited class‐switching and SHM	
		+	IL‐4, GM‐CSF	Class‐switching and increased serum IgG		
		+	IL‐6 (BR6)	Class‐switching and increased serum IgG		
	BLT	+	–	–	Low serum human immunoglobulin	27
		+	IL3, SCF, GM‐CSF (NSG‐SGM3)	Class‐switching and increased serum IgG	Limited class‐switching and SHM	
T cells	huPBL	+	–	Donor immune repertoire transfer	Uniform T‐cell activation due to MHC mismatch	19, 28
					Rapid development of GvHD	
		+	b2m−/−, HLA‐K^b^D^b^ (MHC class I), H2‐Ab1−/− (MHC class II)	Donor immune repertoire transfer	Uniform T‐cell activation due to MHC mismatch	
				Delayed onset of GvHD	Haemochromatosis	
	HIS	+	–	Enables long‐term studies	T‐cell education of murine H2‐restricted T cells	29, 30, 31, 32
					T cells predominantly Th2 polarized	
		+	HLA‐A2.1tg or HLA‐A2/HHD (NSG‐A2), HLA‐DR1tg or HLA‐DR4tg (NSG‐DR1 or NSG‐DR4), NSG‐A2/DR1	Enables study of MHC‐restricted T‐cell responses		
		+	IL12	Improves Th1/Th2 ratio		
	TOM	+	–	T‐cell‐only model for long‐term studies	–	33
	BLT	+	–	Fully functional thymic education, improved mucosal engraftment	–	7, 34
NK cells	huPBL	−	–	–	Absent	
	HIS	−	–	–	Largely absent and impaired functionality	14, 35
		+	IL15 (SRG15)	Improved frequency and functionality		
		+	Flt3L	Improved frequency and functionality		
	BLT	–	–		Largely absent and impaired functionality	
Macrophages	huPBL	−	–	–	Absent	
	HIS	+	–	–	Low numbers of human macrophages	15, 36, 37, 38
		+	M‐CSF, GM‐CSF, IL3 (NSG‐SGM3)	Functional macrophage repopulation	Unphysiological number of Treg cells	
		+	IL3, GM‐CSF, M‐CSF, TPO (MISTRG/MITRG)	Functional macrophage repopulation	Availability, anaemia and short life‐span	
	MOM	+	–	Macrophage‐only model for long‐term studies	B cells still present	39
	BLT	+	–	Increased stability and frequency of myeloid cells		38
Dendritic cells	huPBL	−	–	–	Absent	
	HIS	+	–	–	Low numbers of human dendritic cells	35, 40
		+	Flt3L	Functional dendritic cell responses and higher frequencies	–	
	BLT	+	–	Functional dendritic cell responses and higher frequencies	–	
Neutrophils	huPBL	−	–		Unknown	
	HIS	−	–	–	–	15, 41
		+	IL3, GM‐CSF, M‐CSF, TPO (MISTRG/MITRG)	–	Availability, anaemia and short life‐span	
	BLT	+	–	Increased stability and frequency of myeloid cells	–	38
Eosinophils	huPBL	−	–	–	Unknown	
	HIS	+	–	–	–	37
		+	IL3, GM‐CSF, M‐CSF, TPO (MISTRG/MITRG)	–	Availability, anaemia and short life‐span	15
	BLT	?	–	–	Unknown	
Mast cells	huPBL	−	–	–	Unknown	
	HIS	?	–	–	Unknown	24, 42, 43, 44
		+	SCF	Increased stability and frequency of myeloid cells	–	
		+	IL3, SCF, GM‐CSF (NSG‐SGM3)	Increased stability and frequency of myeloid cells	Unphysiological frequency of Treg cells	
	BLT	+	–	–	Unknown	24
		+	IL3, SCF, GM‐CSF (NSG‐SGM3)	Increased stability and frequency of myeloid cells	Unphysiological frequency of Treg cells	
Erythrocytes	huPBL	−	–	–	Absent	
	HIS	−	–	–	Absent	17
		+	IL15, Flt3L, Epo, IL3	Low numbers reported	–	45
	BLT	−	–	–	Absent	

BLT, bone marrow, liver, thymus mouse; GM‐CSF, granulocyte–macrophage colony‐stimulating factor; GvHD, graft‐versus‐host disease; huPBL, human peripheral blood leucocyte mouse; HIS, human immune system mouse; IL‐4, interleukin‐4; SCF, stem cell factor; SHM, somatic hypermutation; Th1, T helper type 1; Treg, regulatory T cell.

Further developments have shown increased humanization of particular cell lineages among the previously mentioned background strains due to additional cytokine knock‐in, transient protein expression through hydrodynamic delivery and vectors, recombinant protein injection or the expression of additional transgenes. For example, the transient expression of *Epo* and *Il3* displayed increased engraftment levels of human erythrocytes and the transient expression of *Il15* and *Flt3* ligand increases NK cell reconstitution.^45^ Similarly, previous reports demonstrated that depletion of murine macrophages increases engraftment and maturation of human erythrocytes (Table 2).

The SGM3 model developed on the NSG background including additional knock‐ins of human *Il3*, GM‐CSF and stem cell factor, boasts superior as well as stable engraftment of myeloid derived cells and improves B‐cell development, antigen‐specific antibody responses and T regulatory cell development.^38^

Overall, within all humanized mouse models, the degree of chimerism, defined by the proportion of human CD45^+^ cells in the total leucocyte population, varies among background strains, chosen model, HSC donor and between mice. Recently, more advanced models with the goal of dissecting cell‐specific responses to infections have been developed, e.g. myeloid‐only or T‐cell‐only responses.^33^, ^39^, ^46^

## Innate immunity in humanized mice

Myeloid progenitor cells require external stimulatory factors to efficiently differentiate to their subsequent lineages. The main difficulty for this process in humanized mice is the lack of cross reactivity among these factors, rendering the differentiation and maturation of the majority of myeloid derived cells very inefficient *in vivo* (Table 2).

To overcome this, essential cytokines must be added either transiently or through gene knock‐in to establish differentiation of myeloid subsets. This has been a main focus of humanized mouse model development in recent years and led to the development of background strains (SGM3, MISTRG, SRG‐15^14^, ^15^, ^36^) that include human cytokine knock‐in [e.g. interleukin‐3 (IL‐3), IL‐15, GM‐CSF, macrophage colony stimulating factor (M‐CSF)] and so support the engraftment of a wider variety of human cells, particularly myeloid derived cells.

The innate immune cell reconstitution of most humanized mouse models remains substantially lower and does not reflect human immune cell composition. More basic background strains have been shown to support engraftment of classical CD14^high^ CD16^–^ monocyte‐like phenotype in lung tissue of humanized BLT *NOD.Cgscid* ^−/−^ and NSG mice. They also display low levels of CD68^+^ macrophage cell engraftment in the lymphoid and splenic tissue of engrafted mice.^7^ Furthermore, these models are also able to engraft with low levels of different dendritic cell subsets (e.g. CD141^+^, CD303^+^ or CD1c^+^), which exhibit the appropriate maturation status and gene expression profiles following priming of T cells and are sufficiently able to respond to endogenous and exogenous stimulation with Toll‐like receptor ligands and interferon treatment.^47^ Both plasmacytoid dendritic cells and myeloid dendritic cells are able to engraft, with the former creating the majority of the dendritic cell population in peripheral blood, similar to what is observed in humans.^7^, ^47^

The newly developed MITRG and MISTRG models allow for the superior development and more physiological levels of haematopoietic cells essential in innate immune responses, specifically myeloid derived cell subsets such as monocytes and macrophages, but also NK cells.^15^ Both models, MITRG and MISTRG, use immunodeficient *Rag2* ^*−/−*^ *Il2rg* ^*−/−*^ mouse strains and contain gene knock‐ins of *Il3*,* Thpo*,* Csf1* and *Csf2* encoding M‐CSF and GM‐CSF, respectively. In addition to these, the MISTRG model also provides a bacterial artificial chromosome transgene encoding human SIRP*α*, so facilitating the improved engraftment of myeloid subsets^15^ (Table 2).

Furthermore, it has recently been shown that IL‐15 poses a species barrier restricting the development of NK cells.^48^ To allow a more physiological level of engraftment of human NK cells, the SRG‐15 mice were generated. The SRG‐15 incorporates the *Rag2* ^*−/−*^ *Il2rg* ^*−/−*^ background with additional knock‐ins of human IL‐15 and human SIRP*α* driven by the endogenous murine promoter.^14^

Although these improved models display superior engraftment of innate immune cells, their impact on the development and functionality of the subsequent adaptive immune responses is still unknown. Furthermore, many of the gene knock‐ins result in superior humanization. which leads to detrimental effects for the murine host (e.g. rapid development of anaemia^14^).

Despite the presence of myeloid derived cell subsets within many humanized mouse models, the proportion of these cell subsets still remains very unphysiological and so there is still need for more development within all of the models to create an immune cell composition similar to that observed in humans (Table 2).

## Adaptive immunity in humanized mice

One of the main advantages of using humanized mice for *in vivo* research has been the quality of the adaptive immune responses. Each model has its benefits and drawbacks and the choice should be made according to the question to be answered. However, most of the models show both CD4^+^ and CD8^+^ T‐cell engraftment and multi‐organ dissemination.^49^, ^50^

The huPBL model retains the immune memory and antigen specificity from the donor, which may be an advantage; however, this ultimately means that the engrafted human immune system, which consists predominantly of T cells, recognizes and targets the murine cells and ultimately leads to xenogeneic graft‐versus‐host disease.^19^

Despite some major drawbacks in several models due to the lack of human MHC/HLA molecules and the subsequent reactivity against murine MHC molecules, there have been several ways to overcome this, e.g. by introducing human MHC class I and II transgenes or by using the more advanced models (e.g. BLT;^29^, ^51^ Table 2).

BLT mice also exhibit functional antigen‐specific CD4^+^ and CD8^+^ T‐cell responses, but despite this, these cell populations are often biased to display a more naive phenotype, expressing high levels of CD45RA, CD27 and CCR7.^7^, ^52^

The introduction of both the heavy‐chain and light‐chain human HLA class I transgenes (NSG‐HLA‐A2/HHD) induces the development of both CD4^+^ and CD8^+^ cells expressing T‐cell receptor‐*αβ* or CD8^+^ cells expressing T‐cell receptor‐*γδ*. The maturation status of CD8^+^ cells is also depicted through their ability to degranulate, production of interferon‐*γ* (IFN‐*γ*) and their expression of either CD45RO or CD45RA, distinguishing memory from naive CD8^+^ cells.^29^ In addition, NSG‐HLA‐A2/DR1 mice engraft with not only functional T‐cell populations displaying T helper type 1 (Th1), Th2 and Th17 phenotypes, but also 15–20% of CD4^+^ and CD8^+^ exhibited a T effector cell phenotype (CD62L^–^ CCR7^–^ HLA‐DR+). These cells display high polyfunctionality with the ability to produce IFN‐*γ*, tumour necrosis factor‐*α*, IL‐4 and IL‐17 alongside both granzyme and low levels of perforin.^30^ It has also been demonstrated that transgenic mice co‐expressing human HLA‐A2 and HLA‐DR4 did not significantly alter the maturation or development of T‐cell populations; but the HLA‐DR4 single knock‐in mice have an improved polyfunctionality and antigen specificity of effector CD8^+^ cells over the HLA‐A2 mice.^31^ Furthermore, HLA‐DR4 mice have been reported to engraft with high levels of CD4^+^ CXCR5^+^ PD1^++^ T follicular helper cells and their subsequent precursors, which are essential in coordinating downstream B‐cell responses. These cells are disseminated efficiently to mucosal surfaces within these mice, including gut‐associated lymphoid tissue and vaginal mucosa within this model.^53^

Despite the engraftment and functionality of the adaptive immune responses in different humanized mouse models, the inability of human cells to cross react with murine cytokines leads to differentiation problems. It was demonstrated that an increase in IL‐4 production, driving GATA3^+^ expression and double‐positive GATA3^+^ T‐bet^+^‐expressing cells among CD4^+^ T‐cell populations is common to all humanized mouse models. This indicates that T‐cell differentiation is driven towards a Th2‐like status, which is not representative of human CD4 or CD8 T‐cell populations. However, the administration of recombinant human IL‐12 can revert this phenotype and down‐regulate GATA3 and increase T‐bet expression in both CD4^+^ and CD8^+^ T cells accompanied by increased IFN‐*γ* production, indicative of a Th1 phenotype.^32^ Furthermore, there is evidence to suggest that there is active CD8^+^ antigen‐specific T‐cell responses, as studies using human immunodeficiency virus (HIV) have shown that the virus accumulates mutations, actively evolving the dominant T‐cell epitopes, indicating that the CD8^+^ cell responses are dynamically working to suppress viral replication in BLT mice^54^ (Table 2).

Furthermore, adeno‐associated virus delivery of human IL‐2 has been demonstrated to increase the engraftment of regulatory T cells in NSG‐BLT mice.^55^ Notably, however, in certain background strains there is already an unphysiologically high proportion of T regulatory cells, which can be undesirable when investigating responses to infections (Table 2).

## Humoral immunity in humanized mice

Key issues arise in the development of humoral immune responses in humanized mice due to many background strains containing the IL‐2 common *γ*‐chain deficiency. This leads to dramatically under‐developed secondary lymphoid tissues (e.g. lymphoid follicles and gut‐associated lymphoid tissues).^56^ Despite many models exhibiting improved B‐cell development or antibody production, there is still a need for more physiological antibody levels, improved B‐cell development, somatic hypermutation and class switching abilities, before humanized mice can be used for vaccine studies (Table 2).

Previous reports show that the engrafted B‐cell population, even in the more advanced models, displays an immature phenotype with high levels of CD10 and CD5 expression.^34^ Certain models demonstrate an improved diversity of B‐cell subsets, displaying both the B1 and B2 phenotype.^57^ It has, however, been demonstrated that class switching in these models is limited but that B1 phenotyped B cells are able to display a VH‐DH‐JH composition similar to that of the CD34^+^ donor cord blood from which the mice engrafted.^57^ Similarly, plasma cell differentiation and the production of IgM, IgA and IgG antibody subtypes is observed, displaying a similar proportion to that observed in humans, but at lower levels than are required for productive antibody responses.^7^, ^58^ Furthermore, it has been reported that HLA‐DR4 mice display increased B‐cell antibody class switching and IgG secretion.^31^ Similarly, injection of recombinant human IL‐4 and GM‐CSF before tetanus toxoid immunization stimulates memory B‐cell clonal expansion, induces improved B‐cell class switching capacity and provokes increased total IgG, IgM and tetanus‐specific IgG responses^25^ (Table 2). Additionally, humanized mouse models have been used for the study of antigen‐specific antibody responses to various infections.^15^, ^59^

The addition of human *Il6* gene knock‐in has demonstrated improved haematopoiesis, class switching and a high frequency of somatic hypermutation.^26^

Due to this, a further advancement of the MISTRG mouse was created, with the addition of the human *Il6* by gene knock‐in, resulting in the new MISTRG‐6 strain.^26^ This new model shows improved B‐cell and malignant plasma cell development, to investigate their effects on lytic bone disease, however, this strain is yet to be used in the context of infectious disease.^60^

## Viral infections in humanized mice

The development of humanized mice has dramatically improved the quality of *in vivo* research for lymphotropic viral infections. The availability of these small animal models for widespread use in infectious disease research allows an inexpensive, convenient alternative model for dissecting human immune responses and host–pathogen interactions to viral infections, eliminating the need for using chimeric viruses or alternative species as models. Recently, their use has also been expanded to other non‐lymphotropic viral infections.

### Human immunodeficiency virus

As one of the most researched viral infection of the past 30 years, HIV remains a key target for vaccine development, anti‐retroviral therapies and basic research to fully elucidate the mechanisms of infection. Due to the specific CD4^+^ T‐cell tropism, the development of humanized mouse models has had a particular impact on HIV research, especially the BLT model. It has been demonstrated that many characteristic indicators of HIV infection are recapitulated in humanized mice including viraemia, CD4^+^ T‐cell depletion,^61^, ^62^ CD8^+^ T‐cell exhaustion and observed up‐regulation of programmed cell death protein 1,^7^, ^63^ rectal and vaginal transmission and gastrointestinal colonization,^8^, ^9^, ^10^, ^11^, ^62^ development of viral latency^64^ and also evaluation of novel antiretroviral treatments and regimens.^64^, ^65^, ^66^ The use of humanized mice within HIV research has emphasized the significance of varying cell subsets in HIV infection and persistence. The use of novel myeloid‐only mice and T‐cell only mice to dissect cell‐specific immune responses has highlighted the relevance of both macrophages and T cells in sustaining HIV infection.^33^, ^39^ The significance of HIV replication in a model with completely absent T‐cell populations allowed the description of HIV persistence in macrophages throughout antiretroviral treatment.^33^, ^39^, ^46^ Furthermore, reactivating persistent HIV in the perivascular macrophages, astrocytes and microglia in the brain of infected humanized mice further highlights the importance of atypical cell subsets in HIV viral persistence.^67^

### Epstein–Barr virus

Epstein–Barr virus (EBV) infection in humanized mice can recapitulate both the lytic and latent phases of infection, stimulate EBV‐induced lymphomas,^68^ create specific CD8^+^ T‐cell response and expansion,^69^, ^70^ NK‐cell‐mediated EBV restriction, activation and plasmacytoid dendritic cell depletion^71^, ^72^ and both latent and lytic EBV antigens can be detected.^73^ Tumorigenesis from EBV infection can develop in both the peripheral blood injection model (e.g. huPBL*scid*) and in the CD34^+^ HSC injection models upon depletion of human cell subsets controlling oncogenesis.^5^, ^27^

### Kaposi's sarcoma‐associated herpes virus

Kaposi's sarcoma‐associated herpes virus (KSHV) transmission studies have been advanced by the BLT humanized mice, due to the increased immune cell presence in mucosal tissues, supporting natural KSHV transmission routes (e.g. oral and vaginal). Both latent and lytic phases of infection are supported and viral transcripts can be detected in tissues.^74^ However, it has also been shown that simpler models, such as the CD34^+^ injected mice, are susceptible to co‐infection with EBV and KSHV – producing genetic expression profiles similar to that observed in primary effusion lymphoma, which is linked to KSHV infection.^75^

### Human cytomegalovirus

As another potent lymphotropic pathogen, aggressively targeting immunocompromised individuals, accompanied by a lack of appropriate *in vivo* models means that human cytomegalovirus (hCMV) research has welcomed humanized mice as an infection model. It was first demonstrated that it is possible to infect humanized mice with hCMV and recapitulate major steps in the viral disease progression, testing novel antiviral treatments, detection of both early and late transcripts following G‐CSF treatment of humanized mice and reactivation of latent hCMV in multiple organs.^76^ Furthermore, as a common nosocomial infection, transmission of hCMV through peripheral blood stem cell transplantation has been replicated in humanized mouse models and viral DNA was detected in bone marrow, spleen and liver.^77^

### Dengue virus

Humanized mice have been validated as a tool to study dengue virus (DENV) infection due to the appearance of a similar symptomatic infection to those seen in patients, including a rash, thrombocytopenia and fever.^78^ Similarly, the presence of anti‐DENV specific antibodies was observed as early as 2 weeks after infection.^79^ Furthermore, the mice can be infected through mosquito bites, simulating natural transmission.^4^ In addition, studies using NSG mice with transient IL‐15 and Flt3 ligand expression via hydrodynamic injection highlight the importance of NK cells during DENV infection and display infected dendritic cells, which bind directly to, and activate NK cells and subsequently prevent DENV infection in monocytes.^80^

### Human T‐lymphotropic virus

Human T‐lymphotropic virus (HTLV) research, much like HIV research, has also benefitted from the development of humanized models, despite the availability of rabbit, rat and non‐human primate models.^81^ Using many background strains including NOD*scid* ^−/−^, SCID^−/−^ or NOG engrafted with HSC or peripheral blood mononuclear cells or using the BLT models, it has been demonstrated that many aspects of HTLV pathogenesis are recapitulated in humanized mice, such as viraemia,^82^ observing both single and multiple HTLV‐1 integrations,^83^ lobulated lymphocyte nuclei, presence of CD4^+^ Tac^+^ leukaemic cells and neoplastic proliferation, depletion of peripheral CD4^+^ T cells, adult T‐cell leukaemia development^84^ and even neuropathological signs such as myelin breakdown.^82^ It was also described that these models are efficient for the testing and development of drugs, e.g. reverse transcriptase inhibitors.^85^

### Adenovirus

Adenovirus infection is also incredibly limited by the lack of *in vivo* models available to study infection. Adenovirus infection in humanized mice has recently been reported to mimic both the adenovirus acute and latent phases of infection.^86^ Humanized NSG‐A2 mice demonstrate asymptomatic infection in 66% of infected mice, yielding only the expression of E1A, which can be detected throughout major organs within humanized mice including lymph nodes, spleen and bone marrow.^86^ Asymptomatic, latent phase infection produces a productive CD8^+^ antigen‐specific T‐cell response and are able to produce IFN‐*γ* upon *ex vivo* stimulation.^86^

In contrast, the acute infection shows an increase in gene expression driven by the late promoter and displays fibrotic liver histology, and increased monocyte and macrophage cell infiltrates alongside haemorrhagic spots in 34% of infected mice. The acute infection generates increased total IgM levels when compared with the latent infection.^86^ This novel infection model creates a much‐needed method of evaluating adenovirus infection course, potential antiviral candidates and treatment regimens.

### Viral haemorrhagic fevers

The recent use of humanized mice for the study of viral haemorrhagic fevers has improved the quality of research for several haemorrhagic fever viruses including Ebola virus, Hantavirus and Crimean–Congo haemorrhagic fever virus. The distinct pathogenesis of these infections can be recapitulated in humanized mice, which can display features such as Ebola virus antigen detection and liver‐specific histological changes and inflammatory cell infiltrates^1^, ^87^ and species‐specific infection with Ebola virus or Reston virus.^88^ In humanized HLA‐A2 mice, Hantavirus infection induces a dramatic decrease in total platelet count and infiltration of lymphocytes into the lungs, and increases weight loss.^89^ Similarly, in Crimean–Congo haemorrhagic fever infection, there is distinct strain variability observed, liver and brain pathological changes, increased polyfunctionality of CD8^+^ T cells and viral RNA observed in the blood and tissues of infected mice.^90^

### Zika virus

Recent publications from Yi *et al*.^91^ described the first humanized mouse infections with Zika virus. NSG‐HLA‐DR4 mice engrafted with a human immune system from an allogeneic DR4^+^ HSC donor are reportedly susceptible to Zika virus infection. These data elucidate that within this model, the main reservoir of the virus focuses primarily on B cells and myeloid‐derived cells, particularly monocytes. In addition to this, a Zika virus prM and E protein encoding vaccine has also been demonstrated to show efficacy in humanized mice and to elicit protein‐E‐specific antibody production alongside neutralizing antibodies, following 6 weeks of immunization.^91^

### Influenza virus

Regardless of the abundance of small animal models available for influenza virus research, vaccine protection, efficacy testing and screening have particularly benefitted from the use of humanized mouse models. It has been described that both the huPBL model and the HIS models are susceptible to influenza virus infection.^92^, ^93^

Humanized NOD*scid* ^*−/−*^ *β2m* ^*−/−*^ mice display CD8^+^ T‐cell expansion in both peripheral blood and tissues, and display antigen‐specific reactivity towards the matrix M1 protein, with high‐affinity epitope binding upon vaccination with a trivalent attenuated live vaccine candidate.^28^ However, this effect appears largely dependent on the myeloid cell engraftment, namely for the interplay with antigen‐presenting cells, particularly dendritic cells.^28^ In addition, reports of humanized HLA‐DR1 mice, transgenic for human MHC class II, created productive CD4^+^ T‐cell responses specific to H5N1 strains of influenza A virus following priming with H1N1 influenza A virus strains, accompanied by recognition from conserved viral epitopes.^92^ It is also important to note that murine cells (e.g. epithelial cells) are also susceptible to influenza virus infection and wild‐type mice are commonly used as a model for influenza. Hence, it is difficult to define where the response originates in the humanized mice due to the cross reactivity with human and murine chemokines, cytokines and cell–cell interactions.^94^

### Respiratory syncytial virus

As for influenza virus, using humanized mice for respiratory syncytial virus (RSV) infections is advantageous due to researching the virus exploiting its natural tropism; however, the virus itself naturally also infects murine cells, so characterizing the correct response can be difficult.^95^ Despite this, humanized mice are still an encouraging tool for the study of RSV infection and potential treatments. It has been demonstrated that, using adeno‐associated viral vectors, humanized NSG mice that transiently express several human cytokines, including IL‐3, IL‐4, IL‐7, IL‐15, GM‐CSF, M‐CSF and B‐cell‐activating factor were susceptible to RSV infection. Furthermore, this model displayed the expected lung pathologies such as peribronchiolar inflammation, neutrophil infiltration and increased mucus production.^41^ These HIS mice displayed lower levels of infection over NSG controls with increased levels of human IL‐1*β* and CCL‐3.^41^ Furthermore, the human IgA production in the sera was elevated and RSV epitope‐specific CD8^+^ splenocytes were isolated following infection and capable of producing IFN‐*γ*.^41^

## The future for humanized mice

In the past 35 years, the progression and development of humanized mouse models has been astounding. The availability of humanized mice has revolutionized the research of many infectious diseases, especially those that were previously restricted because of their lymphotropic nature. However, there is still much need for improved responses and a correct representation of the heterologous human immune cell populations. Main concerns are that the total human cell count is substantially lower than murine cell counts in wild‐type mice, the deficiency in lymphoid structures, species restrictions with Fc receptor identification and the incompatibility of human and murine MHC molecule presentation and recognition. This subsequently affects the immune response elicited, e.g. total antibody or cytokine serum concentrations, positive and negative selection of T cells.

**Figure 2:**
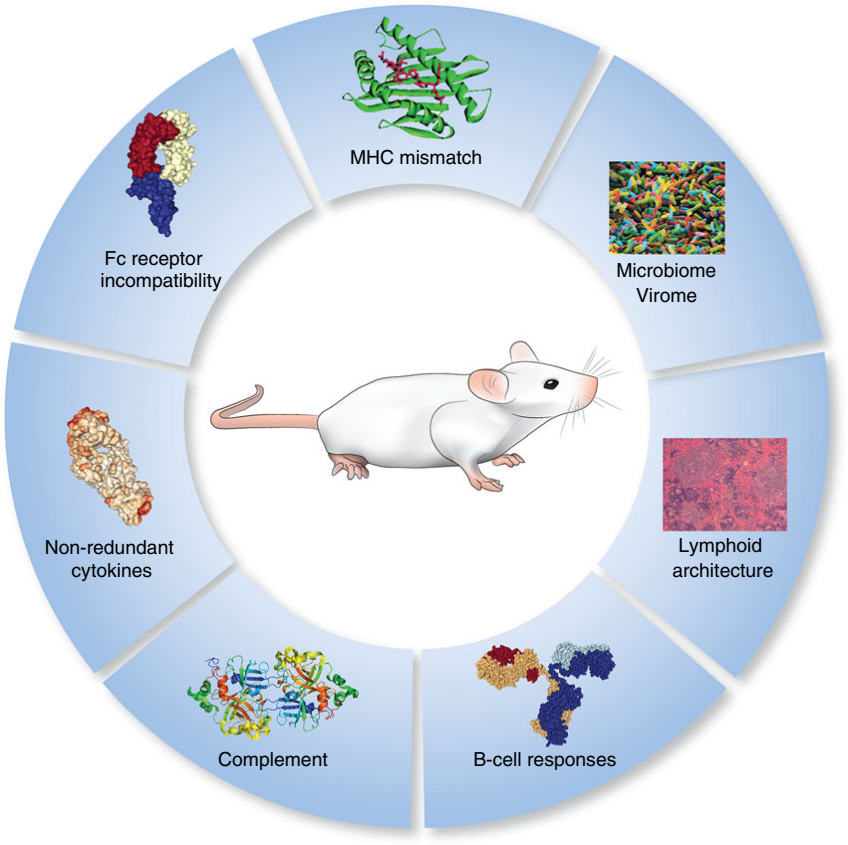
Remaining challenges in the optimization of humanized mice.

Ultimately, several areas of human immunity are significantly under‐represented (Fig. 2), primarily humoral responses and serum antibody diversity. Additionally, the lack of productive VDJ recombination and total serum immunoglobulin concentration preventing efficient execution of the complement pathways and an increased somatic hypermutation.

However, there were substantial improvements in these models in recent years, with the improved engraftment and proficiency of human cells. One of the most notable is the introduction of non‐redundant cytokines (e.g. GM‐CSF, IL‐3), which has increased the efficiency and functionality of the innate immune cells and their response to infection. However, the transgenic mice expressing several of these factors (e.g. MISTRG) have been shown to develop anaemia and only allow short experimental timeframes and so ways of subverting these issues need to be addressed.

Finally, it has also been shown that the microbiome and virome are becoming increasingly relevant for immune responses and can play crucial roles in the course of many infectious diseases, e.g. bacterial translocation from the gut (Fig. 2). Hence, the incorporation of these variables would be an incredibly exciting development in the field.^96^, ^97^

Humanized mouse development is a rapidly evolving field and having a model that fully recapitulates human responses is highly desirable. With a plethora of current models available, optimization of humanization, comparison of different model systems and facilitation of easy access to these models is paramount to their widespread use.

## Disclosure

The authors declare no competing interests.
